# High-throughput sequencing of *Bacillus anthracis* in France: investigating genome diversity and population structure using whole-genome SNP discovery

**DOI:** 10.1186/1471-2164-15-288

**Published:** 2014-04-16

**Authors:** Guillaume Girault, Yann Blouin, Gilles Vergnaud, Sylviane Derzelle

**Affiliations:** 1 University Paris-Est, Anses, Animal Health Laboratory, Bacterial Zoonoses Unit, Maisons-Alfort 94706, France; 2 University Paris-Sud, Institut de Génétique et Microbiologie, UMR 8621, Orsay 91405, France

**Keywords:** *Bacillus anthracis*, Whole genome sequencing, Single nucleotide polymorphism, Molecular typing, Comparative genomics, HRM

## Abstract

**Background:**

Single nucleotide polymorphisms (SNPs) are ideal signatures for subtyping monomorphic pathogens such as *Bacillus anthracis*. Here we report the use of next-generation sequencing technology to investigate the historical, geographic and genetic diversity of *Bacillus anthracis* in France. 122 strains isolated over a 60-years period throughout the country were whole-genome sequenced and comparative analyses were carried out with a focus on SNPs discovery to discriminate regional sub-groups of strains.

**Results:**

A total of 1581 chromosomal SNPs precisely establish the phylogenetic relationships existing between the French strains. Phylogeography patterns within the three canSNP sub-lineages present in France (i.e. B.Br.CNEVA, A.Br.011/009 and A.Br.001/002) were observed. One of the more remarkable findings was the identification of a variety of genotypes within the A.Br.011/009 sub-group that are persisting in the different regions of France. The 560 SNPs defining the A.Br.011/009- affiliated French strains split the Trans-Eurasian sub-group into six distinct branches without any intermediate nodes. Distinct sub-branches, with some geographic clustering, were resolved. The 345 SNPs defining the major B.Br CNEVA sub-lineage clustered three main phylogeographic clades, the Alps, the Pyrenees, and the Massif Central, with a small Saône-et-Loire sub-cluster nested within the latter group. The French strains affiliated to the minor A.Br.001/002 group were characterized by 226 SNPs. All recent isolates collected from the Doubs *department* were closely related. Identification of SNPs from whole-genome sequences facilitates high-resolution strain tracking and provides the level of discrimination required for outbreak investigations. Eight diagnostic SNPs, representative of the main French-specific phylogeographic clusters, were therefore selected and developed into high-resolution melting SNP discriminative assays.

**Conclusions:**

This work has established one of the most accurate phylogenetic reconstruction of *B. anthracis* population structure in a country. An extensive next-generation sequencing (NGS) dataset of 122 French strains have been created that allowed the identification of novel diagnostic SNPs useful to rapidly determine the geographic origin of any strain found in France.

## Background

*Bacillus anthracis*, the causative agent of anthrax, is a spore-forming bacterium found throughout the world. The bacterium mainly affects wild and domesticated herbivores, causing serious, often fatal disease. Spores which are the infectant form of the bacteria can remain in soils for decades before being ingested by grazing animals and later on contaminated browse by blow flies [1]. Consequently, opportunity for accumulating DNA mutations is limited, resulting in relatively little genetic variation within the species.

Single nucleotide polymorphisms (SNPs) are the most common genetic variation found in genomes of all species. It also represents important biologically informative DNA markers extensively used to elucidate deep phylogenetic relationships among worldwide strains, due to their evolutionary stability [2]. By querying a large number of them against collections of diverse strains, a set of canonical SNPs (canSNPs) that define major clades within the *B. anthracis* species has been identified and used for subdividing all isolates into three major lineages (A, B and C) and 13 major sub-lineages or sub-groups [2-5]. Other SNPs that define the A.Br.Ames and A.Br.WNA lineages [6,7] or that lie on various branches of the *B. anthracis* SNP tree [8] have also been published.

As a consequence of the rapid development of technology in the area of high throughput sequencing, extensive genomic sampling is now possible at a reasonable cost. By interrogating nearly every base of the genome, whole genome sequencing is the genotyping tool giving the highest possible resolution, with comparison information spanning from distant phylogenetic relationships to the highest level of subtyping. Identification of thousands of SNPs retrieved from compiled NGS sequences facilitates high-resolution strain tracking and provides the level of discrimination required for microbial forensics. Canonical SNP typing and whole genome comparisons of multiple strains are now becoming the reference method in *B. anthracis* genotyping.

In France, anthrax was a widespread cause of disease in livestock and humans until the beginning of the 20^th^ century. The development of live vaccines for animals by Louis Pasteur (1880–1881), followed by widespread vaccination, had then allowed the numbers of animals affected, and indirectly human cases, to steadily decline. The prevention of new cases and proper carcass disposal with the implementation of knackeries prevented the replenishment of spores in soil in many regions of France and concentrations progressively declined [9]. Today, anthrax is considered as a sporadic disease that continues to cause occasionally outbreaks in livestock. A few cases with one to several dead animals may be recorded annually. However two large outbreaks with several dozens of animals affected still occurred over the past five years in areas where outbreaks had been reported in the past [10].

We have previously reported the canSNP characterization and MLVA typing of a hundreds of strains of *B. anthracis* isolated in France [11]. French strains were assigned into the three B.Br.CNEVA, A.Br.011/009 and A.Br.001/002 canSNP sub-groups and further resolved into about fifty MLVA genotypes (unpublished data). To gain further resolution into the phylogenetic relationships among French isolates, 122 strains have been sequenced using parallel sequencing technology. Comparative genomics, with a focus on SNPs discovery, were then conducted to identify diagnostic SNPs that specifically discriminate regional sub-groups of strains.

## Results

### canSNP genotyping

One hundred and thirty-six “field strains” representative of anthrax activity in France since about half a century (1953–2011) were selected for this study. All strains were first subjected to canSNP typing [11] to place them into a broader phylogenetic context [3]. An additional canSNP assay designed around the A.Br.011 SNP, that further divides the Trans-Eurasian A.Br.008/009 group into two sub-groups (A.Br.008/011 and A.Br.011/009) [4], was also run against the whole collection (data not shown). All French A.Br.008/009 samples (n = 39) were defined as belonging to the A.Br.011/009 sub-group.

### Whole genome sequencing

In order to accurately determine the evolutionary relationships among these strains, 122 isolates were characterized by paired-end whole genome sequencing. We sequenced 67 B.Br.CNEVA-affiliated strains collected in the four areas where this sub-lineage is prevalent (i.e. the Alps (n = 34/38), the Pyrenees (n = 9), the Massif Central region (n = 18/20) and the Saône-et-Loire *department* (n = 6)), 31 A.Br.011/009-affiliated strains isolated throughout the country (n = 31/36) and 24 of the 26 A.Br.001/002-affiliated strains collected from the Doubs *department* (n = 21/22) and the North-East of France (n = 3). An African strain (IEMVT 89) affiliated to the A.Br 005/006 sub-group positioned at the basis of the main A radiation was also whole-genome sequenced for comparison.

The Illumina sequencers produced 4 to 25 million reads per strain after applying the quality filter of the Illumina base-calling pipeline. The filter-passed reads were aligned to the Ames Ancestor reference genome, resulting in more than a 56-fold sequencing depth on average and a genome coverage ranging from 98.9 to 99.9% (Additional file 1: Table S1).

### Extraction of whole strain-specific SNPs among *B. anthracis* strains

**Figure 1:**
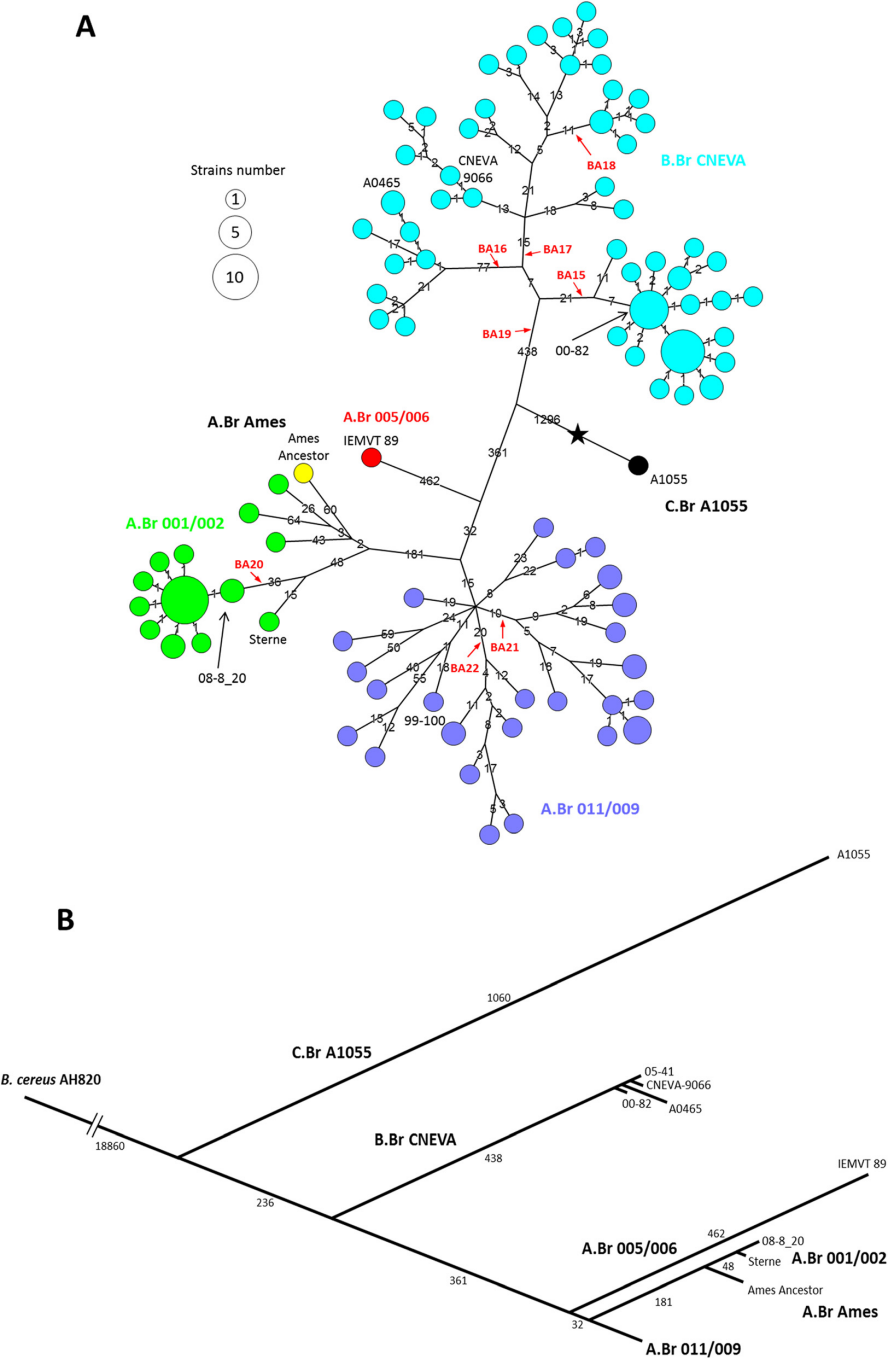
**Phylogeny of 126 *B. anthracis *strains based on whole-genome SNP analysis. A**. Minimum spanning tree based on 3987 chromosomal SNPs. The 3 canSNP groups present in France are color-coded: B.Br CNEVA in light blue, A.Br 011/009 in purple and A.Br 001/002 in green. The African lineage A.Br 005/006 is indicated in red. Positions of the *B. anthracis* Sterne (in green), Ames ancestor (in yellow) and A1055 (in black) strains are also marked. Each circle represents a unique SNP genotype. The diameter of each circle varies according to the number of isolates having the same genotype. The length of each branch is proportional (logarithmic scale) to the number of SNPs identified between strains. Indicated in red are the position and name of the new identified French canSNPs. The star marks the approximate branching point of the *B. anthracis* lineage within the *B. cereus* group. Based on a parsimony approach, the tree size is 4018, i.e. it contains approximately 0.77 % of homoplasia. **B**. Linear phylogenetic tree rooted with the *B. cereus* AH820 strain as outgroup. This figure illustrates the relationship between French and globally diverse *B. anthracis* strains.

Comparative analysis of the genomic sequences was next carried out. Available data for the *B. anthracis* Ames Ancestor, Sterne and A1055 strains were also included. The *B. cereus* AH820 genome was used as outgroup. Figure 1 illustrates the minimum spanning tree (MST) generated by the chromosomal whole-genome SNPs data. A total of 3987 chromosomal SNPs were discovered that precisely positioned French strains within the *B. anthracis* SNP tree. Additional SNPs (128 in pXO1 and 92 in pXO2) were also identified in both plasmids (Analysis of the 122 French strains, including the African strains IEMVT 89, data not shown). Consistent with previous MLVA and canSNP analyses [11], all French isolates were clustered into three distinct groups, i.e. B.Br.CNEVA, A.Br.001/002 and A.Br.011/009. The major B.Br.CNEVA group was separated by a long phylogenetic branch (438 SNPs) to the A and C branches radiation. A total of 1581 SNPs resolved the French population structure at an unprecedented resolution. Strain-specific SNPs can be defined for nearly all particular strains. Moreover, particular genotypes corresponding with geographic regions were observed.

### B.Br.CNEVA phylogenetic analysis

**Figure 2:**
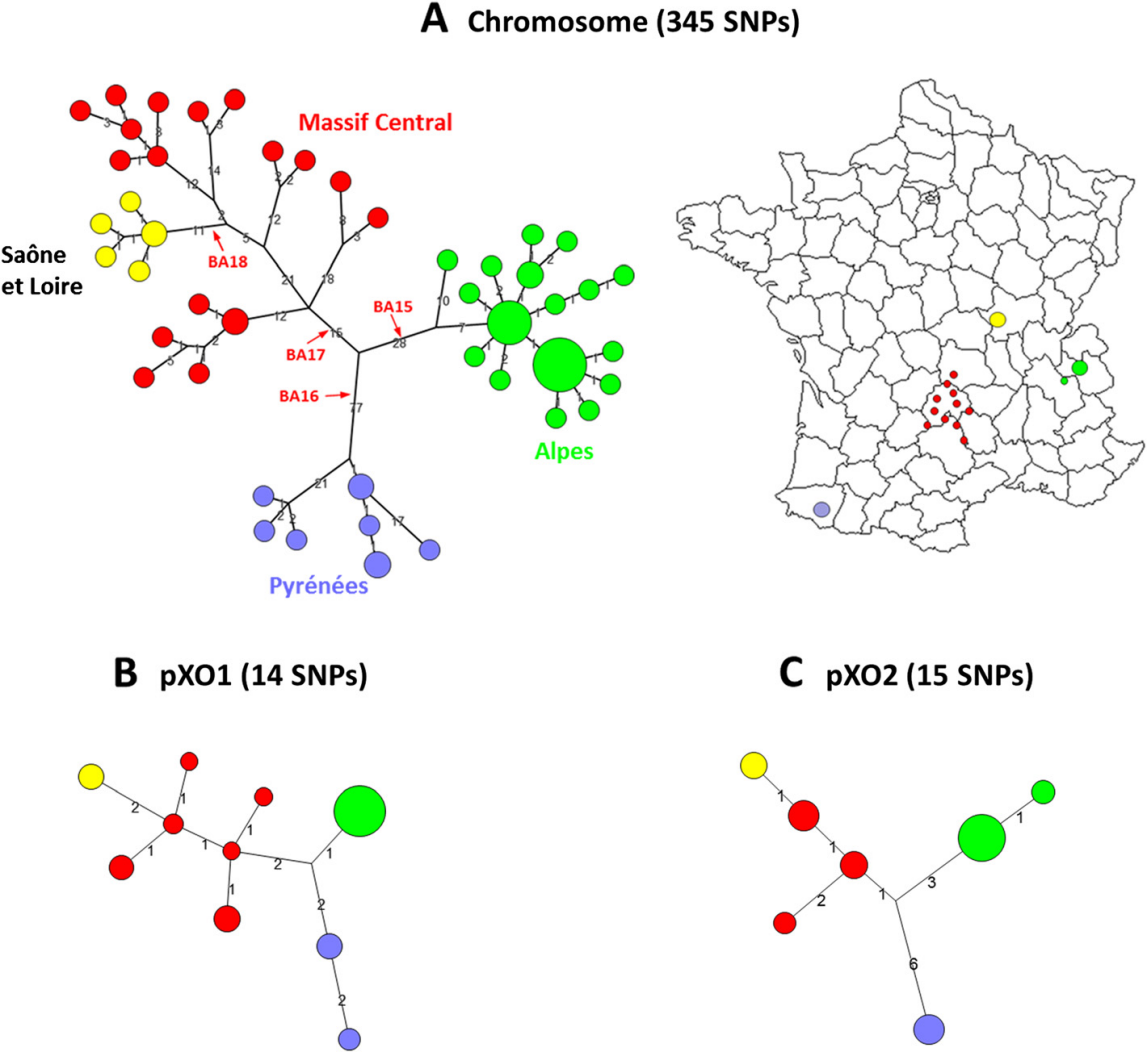
**Minimum spanning tree of 67 French *B. anthracis *strains belonging to the B.Br.CNEVA canSNP lineage.** Data are based on 345 chromosomal SNPs **(A)**, 14 pXO1 SNPs **(B)** and 15 pXO2 SNPs **(C)**. The geographic clustering of the French strains is color-coded: Alps in green (34 strains), Pyrenees in purple (9 strains), Massif Central in red (18 strains) and Saône et Loire *department* in yellow (6 strains). The diameter of each circle varies according to the number of isolates having the same genotype. The length of each branch is proportional (logarithmic scale) to the number of SNPs identified between strains. Indicated in red are the position and name of four French canSNPs described in this study. Based on a parsimony approach, the tree size is 352, i.e. it contains approximately 1.98% of homoplasia. Concerning the plasmids, the tree sizes are 14 and 15 for pXO1 and pXO2, respectively, i.e. it contains no homoplasia.

More than half of the *B. anthracis* isolates from France clustered within the B.Br.CNEVA lineage. We therefore focused on further resolving the positions of its members. The B.Br.CNEVA sub-lineage was characterized by 779 unique chromosomal SNPs, 49 pXO1 SNPs and 49 pXO2 SNPs. Our analysis identified 345 diagnostic SNPs that discriminate between French strains, as well as 29 plasmidic SNPs (Figure 2). Positions of the 67 whole-genome sequenced strains relative to each others are illustrated on Figure 2. A geographic clustering into four sub-groups (Alps, Pyrenees, Massif Central and, nested in this latter, the Saône-et-Loire sub-cluster) was observed using both chromosomal and plasmidic SNPs analysis. Four markers that specifically define one of the newly described geographic sub-groups, as well as one additional SNP shared by all French B.Br.CNEVA genomes, were selected and incorporated into SNP discrimination HRM assays (Table 1).

**Table 1:** French specific canSNPs and primer sequences used for HRM analysis

**canSNP**	Position*	**Target**	**SNP**	**Forward primer (5’-3’)**	**Reverse primer (5’-3’)**	**Product size (bp)**
BA15	126639	Alps (B.CNEVA)	A to G	CCACAAGGTGGAATTATTACTAAAGA	GGTTCACCTGTTTTCGGATCT	80
BA16	3765357	Pyrenees (B.CNEVA)	A to G	GGTGGTTTCGGATATGCACT	AAAGGTGCTGGGGTAGTAAGG	68
BA17	4719494	MC + SL (B.CNEVA)	G to A	TTAGATCTCGTTTTCGGTTCC	CAATGAGTGTACGGCTCCAA	79
BA18	2390832	SL (B.CNEVA)	T to C	CCAGGCAAATACATTGTGGA	TTACAGTCTGTGTTGCCGTTG	73
BA19	2573536	French B.CNEVA	A to G	CATATATTTTCACCTCTTTTATGAACA	GATAAAAGGCTGTCGGATGG	90
BA20	3434997	Doubs (A.001/002)	A to C	AGCGAGCCAATTTTGGAACCGA	AGGCGGGATTGTTGGTGGATGT	63
BA21	3562427	Central NE (A.011/009)	A to C	AGCAAAAAGTCGGCAAAGAA	ACAGAGCTTCCTCCGAACTG	82
BA22	820195	NE (A.011/009)	T to C	AGTGGTGCAATCCCAATTTC	CGCAGCAATATTCGCTATCA	99

*localisation on the Ames Ancestor chromosome (GenBank accession no. AE017334.2). MC: Massif Central; SL: Saône et Loire; NE: North-Eastern.

### A.Br.011/009 phylogenetic analysis

**Figure 3:**
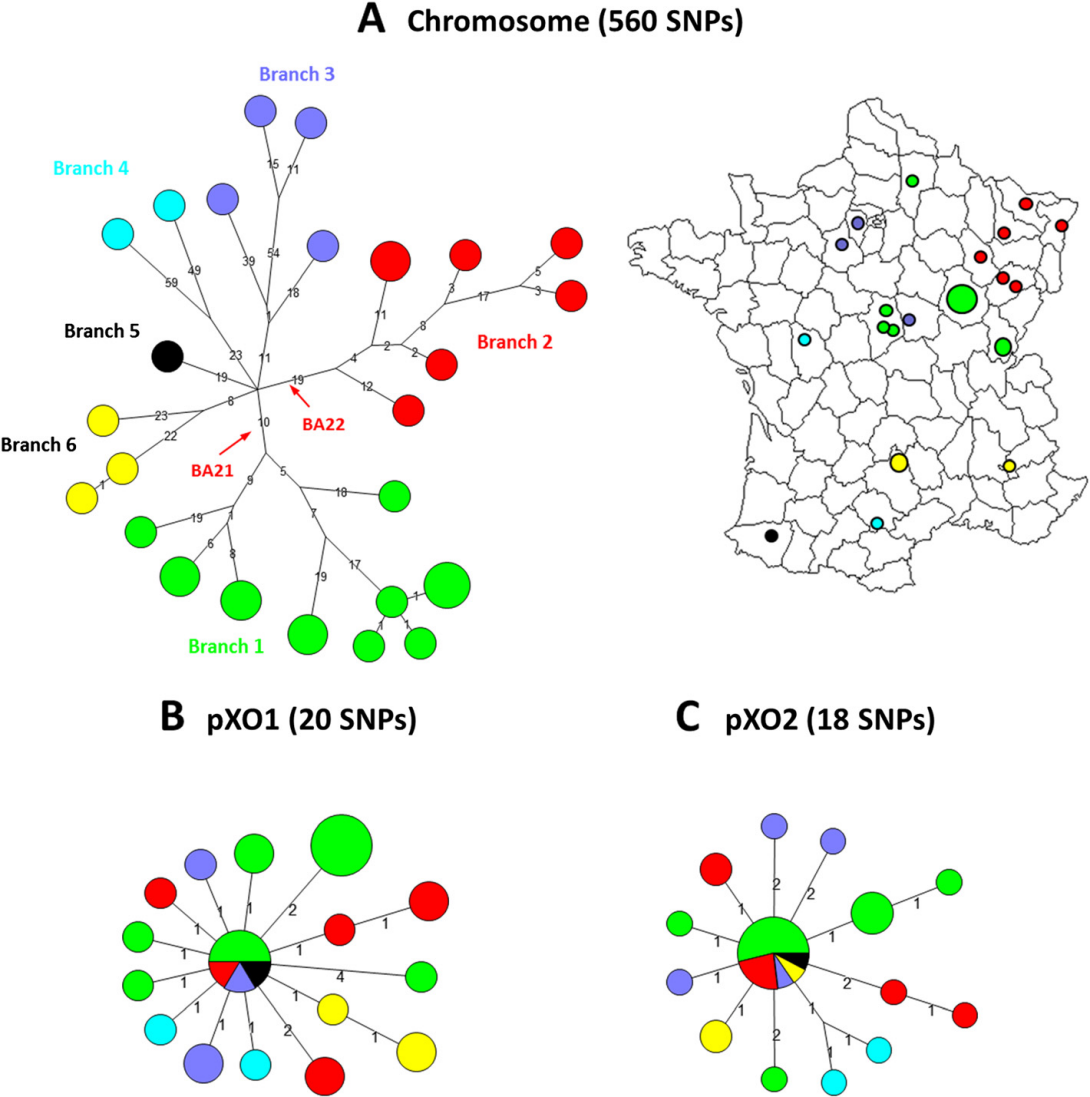
**Minimum spanning tree of 31 French *B. anthracis *strains belonging to the A.Br.011/009 canSNP subgroup.** Data are based on 560 chromosomal SNPs **(A)**, 20 pXO1 SNPs **(B)** and 18 pXO2 SNPs **(C)**. The six resolved branches are color-coded. The diameter of each circle varies according to the number of isolates having the same genotype. The length of each branch is proportional (logarithmic scale) to the number of SNPs identified between strains. Indicated in red are the positions and names for two canSNPs described in this study. NE: North-East, SW: South-West, SE: South-East. Based on a parsimony approach, the tree size is 561, i.e. it contains approximately 0.18% of homoplasia. Concerning the plasmids, the tree sizes are 20 and 18 for pXO1 and pXO2, respectively, i.e. it contains no homoplasia.

Comparison of A.Br.011/009 genomes yielded a total of 574 unique chromosomal SNPs, including 560 SNPs that resolve the sub-group into six distinct sub-branches without any intermediate nodes (Figure 3A). This sub-structure was poorly reflected in both pXO1 and pXO2 SNPs profiles. The 38 Plasmidic SNPs resolved the 31 samples in a higher number of putative branches, without formal clustering (Figure 3B and C). In contrast to the B.Br.CNEVA lineage, a less apparent geographic clustering was also observed within each identified sub-branches (Figure 3A). Branches 1 and 2 include strains isolated in the north-east quarter of France, the outermost samples belonging to branch 2. Branches 3 and 6 are composed of strains isolated around the center (close to Paris) and in the South-East quarter of France, respectively. While the only strain of branch 5 was isolated close to the Pyrenees (South-West), the two samples from branch 4 are from remote geographical regions (West and South). Specific SNPs for the two more relevant branches (1 and 2) were developed into real-time HRM assays (Table 1).

### A.Br.001/002 phylogenetic analysis

Concerning the minor A.Br.001/002 subgroup, sequence analysis identified 226 chromosomal SNPs that differentiate the 24 French strains. Notably, all isolates from the Doubs *department* (21 out 24 samples) formed a single clonal cluster characterized by 44 SNPs (Figure 1). All strains were closely related, differing by a maximum of 2 chromosomal SNPs. Similar SNP patterns were found using both virulence plasmids data (data not shown). Most of them (n = 20) were collected during 17 clustered animal outbreaks occurring in the summer of 2008. An additional strain was isolated in the same area in 2011 and presented the dominant 2008's SNP genotype, suggesting a common point source for these repeated outbreaks. One SNP marker was selected for further testing based upon its specificity for the Doubs strains (Table 1). The three other strains are older “outbreak” samples (1953, 1954 and 1981) isolated in other *departments* of France. They were found to be unrelated to the Doubs recent outbreak isolates. Several specific SNPs differentiating these strains were also found on both plasmids.

### French specific SNP discrimination genotyping assays

We designed eight novel canSNPs discrimination assays based on High-Resolution Melting (HRM)-PCR technology (Table 1). Assays targeted five SNPs identified among the French B.Br.CNEVA genomes, one SNP specific to the A.Br.001/002 strains isolated in Doubs, and two SNPs defining the two large sub-branches 1 and 2 of the A.Br.011/009 sub-group (Figure 1). These diagnostic assays were then successfully validated across the 138 *B. anthracis* strains of our collection. The two expected alternate alleles exhibited distinct melting curves and melting temperatures (Tms), allowing unambiguous grouping of each allele. On average, differences in Tm ranging from 0.5 to 0.8°C were observed between the both allelic states (Table 2). The seventeen non-sequenced genomes of the collection fall on the correct geographical groups (data not shown).

**Table 2:** Melting temperatures (Tm) determined for French-specific canSNP by HRM

**canSNP**	**Region**	**Allele**	**Tm values (°C)**	**Nb strains**
BA15	Alps	G	75.16 ± 0.05	38
	Others	A	74.61 ± 0.07	100
BA16	Pyr	G	79.93 ± 0.04	9
	Others	A	79.39 ± 0.08	129
BA17	MCSL	A	77.27 ± 0.08	26
	Others	G	77.87 ± 0.13	112
BA18	SL	C	79.83 ± 0.10	6
	Others	T	79.13 ± 0.10	132
BA19	France	G	75.84 ± 0.07	73
	Others	A	75.22 ± 0.07	65
BA20	Doubs	C	80.36 ± 0.05	21
	Others	A	79.83 ± 0.06	117
BA21	CNE	C	77.90 ± 0.05	15
	Others	A	77.08 ± 0.08	123
BA22	NE	C	77.50 ± 0.08	10
	Others	T	76.93 ± 0.08	128

Tm values are means ± standard deviation calculated from 138 strains. MCSL: Massif Central and Saône-et-Loire; SL: Saône-et-Loire; Pyr: Pyrenees; CNE: Central North-Eastern ; NE: North-Eastern.

The eight novel canSNPs were further validated *in silico* against all *B. anthracis* genomes (n = 30) available in public NCBI database and Sequence Read Archive (SRA) [12]. The new markers were also screened across a hundred of non-French *B. anthracis* DNAs of diverse origins (described by 10 canSNP typing, unpublished data) to confirm their specificity. The eight assays accurately separated the French strains from the remaining globally diverse genotypes (data not shown).

## Discussion

Comparative whole-genome sequencing offers a powerful way for in-depth characterization of any bacterial pathogen. It also provides an unbiased approach for informative SNPs discovery [13]. In this study, an ultimate picture of the genetic diversity found within the *B. anthracis* population in France has been established. An extensive NGS dataset of 122 autochthonous strains have been created that allowed the phylogenetic linkage of geographically diverse isolates and the identification of novel SNPs signatures useful to rapidly determine the geographic origin of any strain ecologically established in France.

Within the B.Br. CNEVA lineage, which is the most successful group in frequency found in France, a clustering concordant with the geographical origin of each strain was highlighted. This phylogeography pattern is consistent with a single introduction of this lineage in the country, followed by dispersal of the ancestral population toward West and South, with progressively derived local populations of *B. anthracis* strains. Ecological establishment and *in situ* differentiation within three regions with environmental conditions particularly favorable for the survival of the spores (i.e. grasslands made of pastoral valleys) are observed: The Alps, The Pyrenees and the Massif Central, with further derived sub-clade in Sâone-et-Loire *department* (discriminative canSNPs BA15 to BA18). The French B.Br.CNEVA lineage forms a large cluster distinct from similar strains isolated elsewhere in Europe (canSNP BA19). It is worthwhile to mention that A strains are adapted to more diverse environments than B strains, which are restricted to more narrow environmental conditions [14]. This trend is reflected on a global scale. A strains have experienced a global dissemination and population explosion, while both B lineages are uncommon in much of the world and appear to be only successful locally or regionally [3].

In contrast to the B.Br.CNEVA lineage, French strains affiliated to both A.Br.001/002 and A.Br.011/009 groups appeared to be more disparate and less related to one another. Several distinct introductions and subsequent *in situ* differentiation of both canSNP groups in France could therefore not be excluded. One of the more remarkable findings from the whole genome SNP analysis was the discovery of a complex pattern of six phylogenetic sub-branches (composed of up to fifteen strains) within the A.Br.011/009 isolates collected in France. The TEA group (A.Br.008/009) is a cluster that predominates throughout Europe, the Middle East and the Western most Chinese province of Xinjiang [3,15]. The identification of six sub-branches, with some geographic clustering (canSNPs BA21 and BA22), indicates an extensive history for the particular A.Br.011/009 TEA sub-group in France that could be linked to an expansion into many novel environments throughout the country or a combination of possibly repeated introduction and infections. Similar pattern of loosely related strains (≈100 SNPs) was also observed for the A.Br.001/002 canSNP group, except for strains isolated from the Doubs *department* (canSNP BA20). All but one of these Doubs specimens were isolated from a single episode (involving 17 clustered outbreaks) associated with the death of 39 animals in 2008 [16]. Only a short evolutionary time period, i.e. very few SNPs, separate all Doubs isolates, reflecting recurring outbreaks in this specific area. Further comparisons with European strains affiliated to the same canSNP groups are, however, need for drawing sound conclusions on the evolution and natural history of both A.Br.011/009 and A.Br.001/002 canSNP groups in France.

Although other relationships are topologically similar to MLVA predictions, some discrepancies exist between whole-genome SNP- and MLVA-based phylogenetic reconstructions (data not shown). There result from homoplasy coupled with weaker character support in the VNTR system. SNPs are more stable and definitive markers than VNTRs. In the MLVA-31 minimum spanning trees, for example, the French B.Br.CNEVA strains are grouped into three clonal complexes (CCs) and a few singletons unrelated to others strains from close geographical origins (Thierry et al., unpublished data). Most of the strains from The Alps, The Massif Central and part of the Saône-et-Loire samples were clustered in a single, large clonal complex (CC1). Strains from the Pyrenees formed a distinct clade (CC2), as well as half of the Saône-et-Loire strains (CC3). The Saône-et-Loire isolates were erroneously separated within two different clusters based on difference in up to four VNTR loci between CC1 and CC3. The discriminative power of the 31 VNTR markers is also more limited to resolve strains from a specific region or outbreak, as illustrated by the A.Br.001/002 strains collected in the Doubs 2008 and 2011. While MLVA31 differentiated five genotypes, nine distinct SNPs profiles could be resolved by whole-genome SNPs scanning. Furthermore, the latter clearly demonstrated that all Doubs strains are genetically related and differs by only one to two SNPs throughout the whole genomes. SNP-based phylogenetic reconstruction of *B. anthracis* population structure is more reliable. Whole genome comparison and canSNPs genotyping represent the future of molecular epidemiology analysis.

## Conclusions

In this study, an ultimate picture of the genetic diversity found within the *B. anthracis* population in France has been established. The knowledge derived from this work is extremely useful for future outbreak investigations, not only due to the specific genomic information that facilitates detailed comparisons between different bacterial isolates, but also because the data can be used to add unprecedented resolving power to current molecular typing tools. Using HRM, eight novel canSNP assays that narrowly defined the three *B. anthracis* genetic sub-lineages found in France were developed. As a consequence, whether an index strain is representative of ecologically established strains from France or have been imported from other areas can be rapidly determined.

## Methods

### Bacterial strains and biosafety procedures

A total of 136 *B. anthracis* strains belonging to B.Br CNEVA (n = 73), A.Br 011/009 (n = 36), A.Br 001/002 (n = 26) and A.Br 005/006 (n = 1) were used in this study. All but one strain were isolated in France. It included nine old reference strains from the Pasteur Institute’s CIP collection (e.g. 17JB, CIP 53.169, CIP 74.12, CIP 77.02, CIP 81.89, CIP A204, CIP A205, CIP A206 and CIP A211), 126 strains collected during animal or human anthrax outbreaks (mostly from bovine origin) that have occurred in France over the past 31 years (1982–2013), among which the previously sequenced CNEVA 9066 and A0465, and one African strain (IEMVT 89–1620) affiliated to the A.Br.005/006 sub-group. *B. anthracis* species was confirmed by bacteriology and PCR. All *B. anthracis* manipulations were performed in a biosafety level 3 laboratory using class II type A2 biosafety cabinet.

### DNA extraction

Genomic DNAs were obtained from vegetative cells grown at 37°C on 5% horse blood agar plates. DNA was purified using the QIAGEN® Genomic-tip 100/G columns and QIAGEN® Genomic DNA Buffer Set. Briefly, bacterial colonies were harvested by scraping the agar surfaces from 16 to 18 h-old Petri dishes. Cell pellet was resuspended in 3.3 ml of Buffer B1 containing 7 μl of RNase A (100 mg/ml, QIAGEN) and incubated at 37°C for 45 min with 350 μl of lysozyme (100 mg/ml, Roche) and 100 μl of proteinase K stock solution (QIAGEN). Following addition of 1.2 ml of Buffer B2, DNA lysate was further incubated at 50°C for 1 h. Particle-free sample was next applied to the equilibrated QIAGEN Genomic-tip and DNA purification on anion-exchange resin processed according to the manufacturer’s recommendations. After isopropanol precipitation, genomic DNA was resuspended in 400 μl of 10 mM Tris HCl (pH 8) for at least 2 h at 50°C.

DNA solutions were transferred to a 0.22 μm sterile Ultrafree-MC spin filter (Millipore) and centrifuged for 2 min at a maximal speed of 8000 × g to ensure the complete removal of live forms of *B. anthracis* from DNA. Viability testing was systematically performed before DNA was taken out of the BSL-3 facility. An aliquot of each DNA preparation (a quarter) was spread on Petri dishes and grown at 37°C for 24 h.

### Draft whole genome sequencing (WGS) and data analysis

In this study, 122 French strains belonging to 3 sublineages (A.Br 001/002 (n = 24), A.Br 011/009 (n = 31) and B.Br CNEVA (n = 67)) were sequenced at the IMAGIF sequencing platform (Imagif, Gif sur Yvette, France) [17]. Isolates were subjected to paired-end whole genome sequencing on either the Illumina Genome Analyzer IIx instrument (16 samples, paired-end data of 2x75pb) or the Illumina HiSeq2000 platform (107 samples, paired-end data of 2x100pb) (Illumina Inc., San Diego, CA, USA). Genome coverage of at least 57× was obtained (Additional file 1: Table S1, average depth ranging from 57× to 433×). The number of reads that passed Illumina quality filters varied from 4.5 to 26.8 million.

Ames Ancestor [GenBank:AE017334.2] was used as the reference genome for assembly. Ames Ancestor plasmid pXO1 [GenBank:AE017336.2] and pXO2 [GenBank:AE017335.3] were used as references for plasmids assembly. Short reads data sets were exported on the FastQ format and mapped to the Ames Ancestor genome and both pXO1 and pXO2 plasmidic sequences using BioNumerics version 6.6 (Applied Maths, Belgium) and Power assembler module asking for a similarity of at least 90%. A set of SNPs was deduced for each genome sequence data using BioNumerics Chromosome Comparisons module. Individual lists were compiled by using an in-house Python script, and data filtered to remove SNP positions at which one or more isolate displayed an ambiguous residue call or missing data. Ribosomal operons and VNTR loci were also excluded from the analysis, so as contiguous SNPs (in a 10pb window). The list of canSNP positions is provided in Table 1.

The draft genome sequences of one representative French strains affiliated to the A.Br.001/002 (08-8_20, Doubs) [NCBI: JHCB00000000], A.Br.011/009 (99–100, Branch 3) [NCBI: JHDR00000000] and B.Br.CNEVA (00–82, Alps) [NCBI: JHDS00000000] have been deposited in the NCBI database. The whole genome sequences for *B. anthracis* strains CNEVA-9066 (B.Br.CNEVA, Massif Central) [NCBI: NZ_AAEN00000000.1], A0465 (B.Br.CNEVA, Pyrenees) [NCBI: NZ_ ABLH00000000.1], Ames ancestor (A.Br.Ames) [NCBI: NC_ 007530.2], Sterne (A.Br.001/002) [NCBI: NC_005945.1] and *B. cereus* AH820 (outgroup) [NCBI: NC_011773.1] can be found in the NCBI microbial genome website at http://www.ncbi.nlm.nih.gov/.

### Whole genome phylogenetic analysis

A minimum spanning tree was drawn in BioNumerics by using the filtered whole genome sequencing SNP data as input. The tree was rooted by using the *B. cereus* AH820 strain as outgroup. *B. cereus* AH820 resides very close to the *B. anthracis* cluster using multilocus sequence typing [18]. Nodes were numbered by BioNumerics. The canSNPs along the three branches leading to the French strains were identified from whole genome sequencing data by searching for SNPs with allelic states shared only by these different subgroups.

### SNP discrimination assays by HRM

We designed High Resolution Melting (HRM) assays for eight French-specific SNPs using Primer 3^+^ software [19]. The positions of these SNPs in the Ames Ancestor genome [GenBank: AE017334.2] and the primers sequences used are listed in Table 1.

Amplification was performed on the ViiA7™ Real-Time PCR System (Life Technologies) using the LightCycler® 480 High Resolution Melting Master Mix (Roche Diagnostics). The reaction mixture consisted of 0.2 μM of each primer, 1 × LightCycler® 480 HRM master mix and 2.5 mM MgCl2 in a 10-μl final volume. The following parameters were used: 10 min at 95°C were followed by 40 cycles consisting of 10 s at 95°C, 10 s at 58°C and 20 s at 72°C. Samples were next heated to 95°C for 30 s, cooled down to 65°C for 1 min and heated from 65°C to 88°C at a rate of 1°C/s with 25 acquisitions/°C. HRM data were analyzed by the ViiA7™ Software (version 1.2.1).

### Availability of supporting data

The ANSES Genome Shotgun project has been deposited at DDBJ/EMBL/GenBank under the BioProject number PRJNA242332, with accession numbers JHCB00000000 (A.Br 001/002, 08-8_20, Doubs), JHDR00000000 (A.Br 011/009, 99–100, Branch 3) and JHDS00000000 (B.Br.CNEVA, 00–82, Alps).

Phylogenetic trees data are available from the Dryad Digital Repository (http://datadryad.org/) with the following identifier: http://doi.org/10.5061/dryad.rc6m9.

## Abbreviations

DNA: Deoxyribonucleic acid; SNP: Single nucleotide polymorphism; canSNP: Canonical single nucleotide polymorphism; VNTR: Variable-number tandem repeat; MLVA: Multiple-locus variable-number tandem repeat analysis; WGS: Whole-genome sequencing; NGS: Next generation sequencing; PCR: Polymerase chain reaction; HRM: High resolution melting; CC: Clonal complex; NCBI: National center for biotechnology information; TEA: Trans-Eurasian.

## Competing interests

The authors declare that they have no competing interests.

## Authors’ contributions

GV and SD conceived the study. GG and SD performed the experiments, analyzed the data and wrote the paper. YB developed scripts used for whole-genome SNP analysis. All authors read and approved the final manuscript.

## Supplementary Material

Additional file 1: Table S1 Strains used in this study and NGS data. Click here for file
